# Water‐Assisted Exfoliation of HfO_2_‐Based Membrane for Flexible Robust Ferroelectric Synaptic Transistors

**DOI:** 10.1002/advs.202523654

**Published:** 2026-01-28

**Authors:** Han Zhang, Miao Zeng, Jingxin Chen, Lei Wang, Sai Jiang, Qian Cao, Xuebo Li, Zekun Hou, Chun Shen, Yulin Gan, Ning Fang, Zhaoliang Liao, Ziyao Zhou, Lin Hao

**Affiliations:** ^1^ The Materials and Electronics Research Center Changzhou University Changzhou Jiangsu China; ^2^ Anhui Provincial Key Laboratory of Low‐Energy Quantum Materials and Devices High Magnetic Field Laboratory HFIPS Chinese Academy of Sciences Hefei Anhui China; ^3^ National Synchrotron Radiation Laboratory School of Nuclear Science and Technology University of Science and Technology of China Hefei China

**Keywords:** artificial synapses, flexible oxide films, HfO_2_–ZrO_2_ heterostructures, reservoir computing

## Abstract

HfO_2_‐based thin films possess broad application potential in semiconductors, non‐volatile memory, and neuromorphic computing owing to their high dielectric constant, excellent ferroelectricity, and environmental robustness. However, an environmentally friendly strategy for synthesizing flexible HfO_2_‐based films remains lacking. In this work, by incorporating a BaTiO_3_/Hf_0_._5_Zr_0_._5_O_2_/BaTiO_3_ (BTO/HZO/BTO) sandwiched architecture together with a Sr_4_Al_2_O_7_ sacrificial layer, a freestanding HfO_2_‐based heterostructure can be detached simply by applying pure water. The resulting film exhibits high film quality and stable ferroelectric behavior, along with pronounced flexibility that enables repeated transfer onto diverse substrates. Furthermore, the synthesized freestanding film is employed to construct a ferroelectric field‐effect transistor that successfully emulates synaptic functionalities, highlighting its potential for low‐power neuromorphic hardware. This work provides a viable strategy for developing high‐performance artificial synapses based on flexible oxide heterostructures.

## Graphical abstract

A flexible freestanding HfO_2_‐based ferroelectric membrane is achieved via a water‐assisted exfoliation technique using a Sr_4_Al_2_O₇ sacrificial layer. The BaTiO_3_/Hf_0.5_Zr_0.5_O_2_/BaTiO_3_ heterostructure maintains robust ferroelectricity and exhibits reliable synaptic plasticity. It successfully performs low‐power reservoir computing tasks, including ECG arrhythmia classification and handwritten digit recognition. This work provides a viable strategy for developing high‐performance, flexible neuromorphic devices based on ferroelectric oxides.

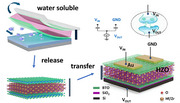

## 1 Introduction

The emergence of conductive polymers as a novel class of materials in the early 21^st^ century marked a milestone in the development of flexible electronics [1, 2] Over the following decades, flexible materials have been introduced into electroluminescent devices, solar cells, organic transistors and field‐effect transistors, polymer dot memory, and organic sensors [3, 4, 5, 6, 7]. During this period, conventional inorganic compounds, which generally lack intrinsic elasticity, received limited consideration in flexible applications [5, 8]. Progress accelerated only after 2019, when freestanding perovskite films such as BaTiO_3_ (BTO) and PbZrO_3_ were successfully fabricated using pulsed laser deposition (PLD) in combination with hydrolytic or support‐layer‐assisted release techniques [9, 10]. This breakthrough enabled inorganic compounds to emerge as viable candidates for flexible electronics. In 2023, researchers at the University of Science and Technology of China further refined the method, extending it to a wider family of inorganic flexible materials [11] Compared with organic materials, oxide thin films typically offer superior durability, thermal stability, and compatibility with micro‐/nano‐fabrication processes such as photolithography and etching, making them attractive for flexible micro‐electronic device engineering [5, 12]

**FIGURE 1:**
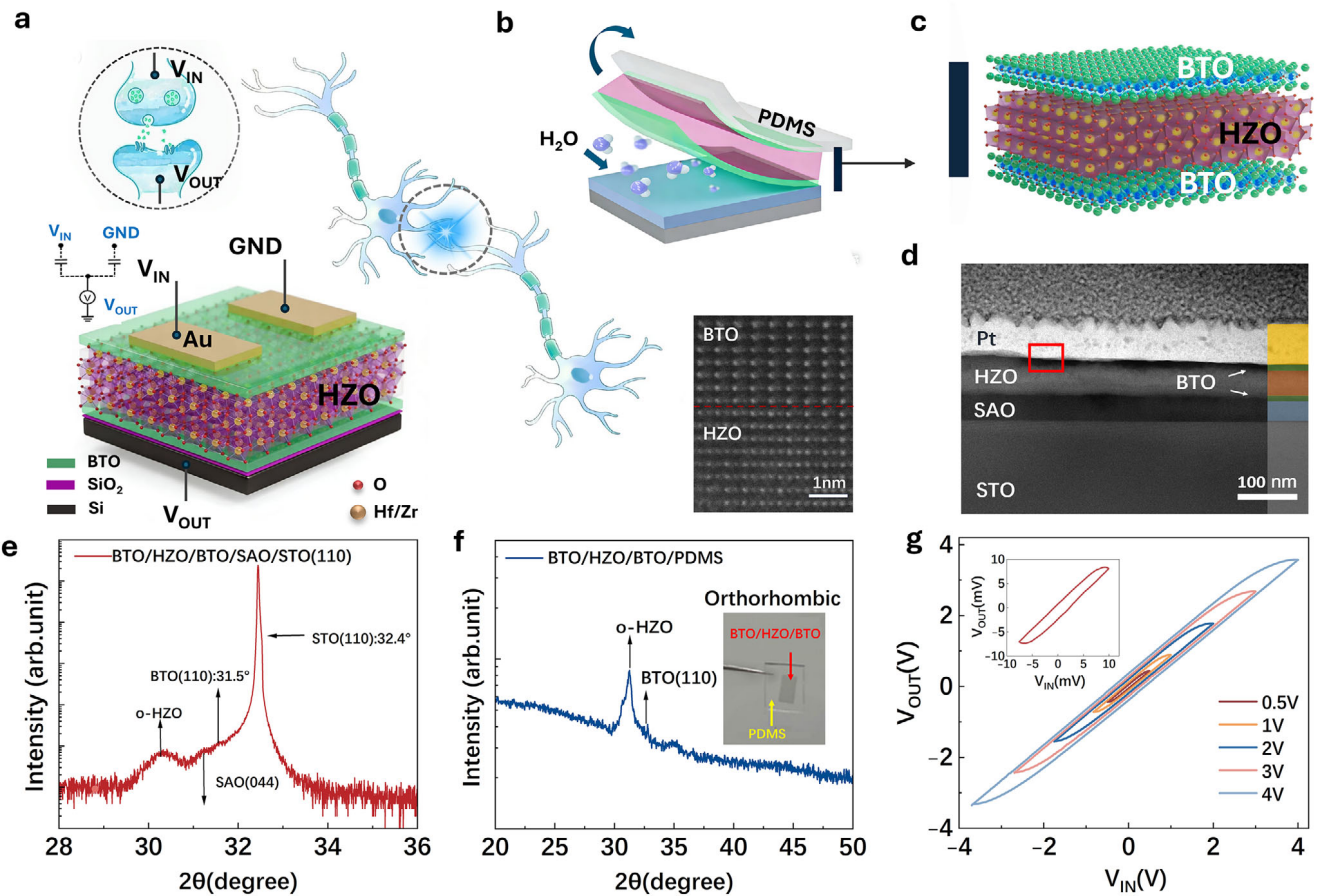
Structure and characterization of the synaptic transistors. (a) Schematic of the freestanding HZO capacitive synapse. (b,c) Fabrication process of the freestanding HZO film. (d) HAADF‐STEM image of the BTO/HZO/BTO/SAO/STO stack showing a clean HZO/BTO interface. (e‐f) XRD patterns of the heterostructure on the substrate (e) and after release onto PDMS (f); inset in (f) shows an optical image of the freestanding membrane (2 × 5 mm). (g) V_OUT_–V_IN_ hysteresis loops measured as the driving voltage decreases from ±4 to ±0.5 V. Inset: close‐up view from −0.01 to 0.01 V.

Among non‐organic ferroelectric/dielectric systems, HfO_2_‐based thin films are particularly promising for semiconductor memory and neuromorphic architectures because of their high‐κ dielectric properties, stable ferroelectricity, and resilience under harsh operating environments [13, 14, 15, 16]. Neuromorphic devices emulate biological synapses to perform efficient and low‐power signal processing [17, 18, 19, 20]. However, the integration of HfO_2_‐based materials with flexible substrates is hindered by the intrinsic trade‐off between ferroelectric stability and the mechanical compliance required at ultrathin thicknesses [21, 22, 23]. A recent study demonstrated that Zn‐doped HfO_2_ deposited on a La_0_․_67_Sr_0_․_33_MnO_3_ (LSMO) layer could be partially released to form a flexible ferroelectric film after chemical removal of the sacrificial layer [24]. Nevertheless, acid etching is generally undesirable in device fabrication, and the released HZO layer alone offers limited flexibility, preventing reliable multistep transfer [25, 26]. Meanwhile, the solution‐based deposition of Zn‐doped HfO_2_ often reply on toxic organic precursors and can only be used to synthesize polycrystalline samples [27]. Thus, fabrication of a freestanding HfO_2_‐based film that maintains ferroelectricity while exhibiting genuine flexibility remains an open challenge. Here, we demonstrate a strategy that overcomes these limitations. By inserting ultra‐flexible BTO thin buffer layers, which impart tensile strain to stabilize the ferroelectric phase, and employing a water‐soluble Sr_4_Al_2_O_7_ (SAO) sacrificial layer for its high dissolution rate and epitaxial compatibility. This approach allows for the complete release of the BTO/HZO/BTO heterostructure film using only deionized water (Figure 1b,c). Meanwhile, the BTO buffer layers impart exceptional bendability, enabling smooth transfer to arbitrary host substrates [9, 28]. The harvested film is also integrated into a ferroelectric field‐effect transistor (FEFET) to highlight its feasibility for flexible oxide‐based synaptic devices. As shown schematically in Figure 1a, the top gate receives external voltage stimuli analogous to a presynaptic terminal, while the channel behaves as the postsynaptic response, functionally reproducing synaptic signal modulation.

## 2 Results and Discussion

### 2.1 Fabrication and Structural Characterization

HfO_2_ normally crystallizes in a monoclinic (m‐phase, P2_1_/c) structure under ambient conditions and undergoes phase transitions to tetragonal and cubic symmetries as temperature approaches 3000 K [29, 30, 31]. A metastable orthorhombic (o‐phase) configuration, which breaks inversion symmetry, gives rise to ferroelectricity. Stabilization of the o‐phase can be achieved by tuning deposition conditions or by aliovalent doping [15, 32, 33]. Partial substitution of Hf by Zr significantly enhances ferroelectric behavior relative to pristine HfO_2_.Studies on Hf_1__−__x_Zr_x_O_2_ confirm that ferroelectricity is optimized near *x* = 0․5, corresponding to the Hf_0_․_5_Zr_0_․_5_O_2_ (HZO) composition employed in this work [34, 35, 36].

By depositing directly on top of the water‐soluble SAO sacrifice layer, HZO is seen to stabilize in its rhombohedral phase (r‐HZO) probably due to the moderate lattice mismatch [37], although the r‐HZO also exhibit some extend of ferroelectricity, its metastable nature may pose challenges for long‐term endurance in practical applications [38]. Further, the film also exhibits weak flexibility which renders it easily shattered into pieces during the water‐assisted transfer process (See Figure S1 in the Supplementary Information for details). To address both of the above issues, BTO buffer layers were introduced where an orthorhombic ferroelectric phase of HZO was eventually stabilized with greatly enhanced flexibility at the same time. Figure 1b,c presents the stacked heterostructure consisting of a 40 nm HZO layer confined between two 10 nm BTO layers that provide improved mechanical resilience and a strain‐inducing template. A 35 nm water‐soluble SAO sacrificial underlayer was first deposited on the SrTiO_3_ (STO) substrate prior to heterostructure growth. Thin‐film synthesis was carried out using PLD under real‐time monitoring by reflection high‐energy electron diffraction (RHEED). Interfacial crystallinity was then examined by scanning transmission electron microscopy (STEM). A representative cross‐sectional image in Figure 1d reveals a coherent and sharply defined epitaxial interface. The Energy Dispersive Spectroscopy (EDS) shown in Figure S3 confirms no inter‐diffusion of the elements.

The heterostructure is subsequently immersed in pure water to dissolve the SAO sacrificial layer using a PDMS‐supported transfer method (Figure 1b). Figure 1c displays the released BTO/HZO/BTO film, whose XRD patterns (Figure 1e,f) exhibit a clearly resolved [111] peak of the o‐phase HZO, confirming a successful transfer process. The freestanding heterostructure can be repeatedly laminated onto rigid or flexible substrates and integrated with various 2D systems.

### 2.2 Ferroelectric and Synaptic Properties

For demonstration, a BTO/HZO/BTO field‐effect transistor (FET) was fabricated via photolithography on n‐type silicon serving as the universal back‐gate (Figure 1a). To verify ferroelectric switching, the input signal V_in_ modulates the capacitive polarization of the HZO dielectric layer, inducing ferroelectric inversion and a corresponding change in V_out_. Such behavior aligns with the reservoir computing (RC) framework, requiring rapid, low‐power temporal computation. The device underwent voltage sweeps with amplitudes between ±0.5 and ±4 V, as presented in Figure 1g. A pronounced hysteresis over the entire voltage range confirms robust ferroelectricity, with no performance degradation when monitored over a 20‐day period (Figure S4c in the Supplementary Information). Moreover, the inset scans at 10 mV also display well‐defined hysteresis, indicating operation at exceptionally low energy without significant loss in performance. The membrane was also transferred to a Indium Tin Oxide/polyethylene terephthalate (ITO/PET) flexible substrate and mounted it to a bending apparatus (see Supplementary Note S6 for details). The results suggest the membrane to exhibit no observable fractures with stable electrical signal down to R = 2cm, where R is the bending radius, confirming flexibility.

**FIGURE 2:**
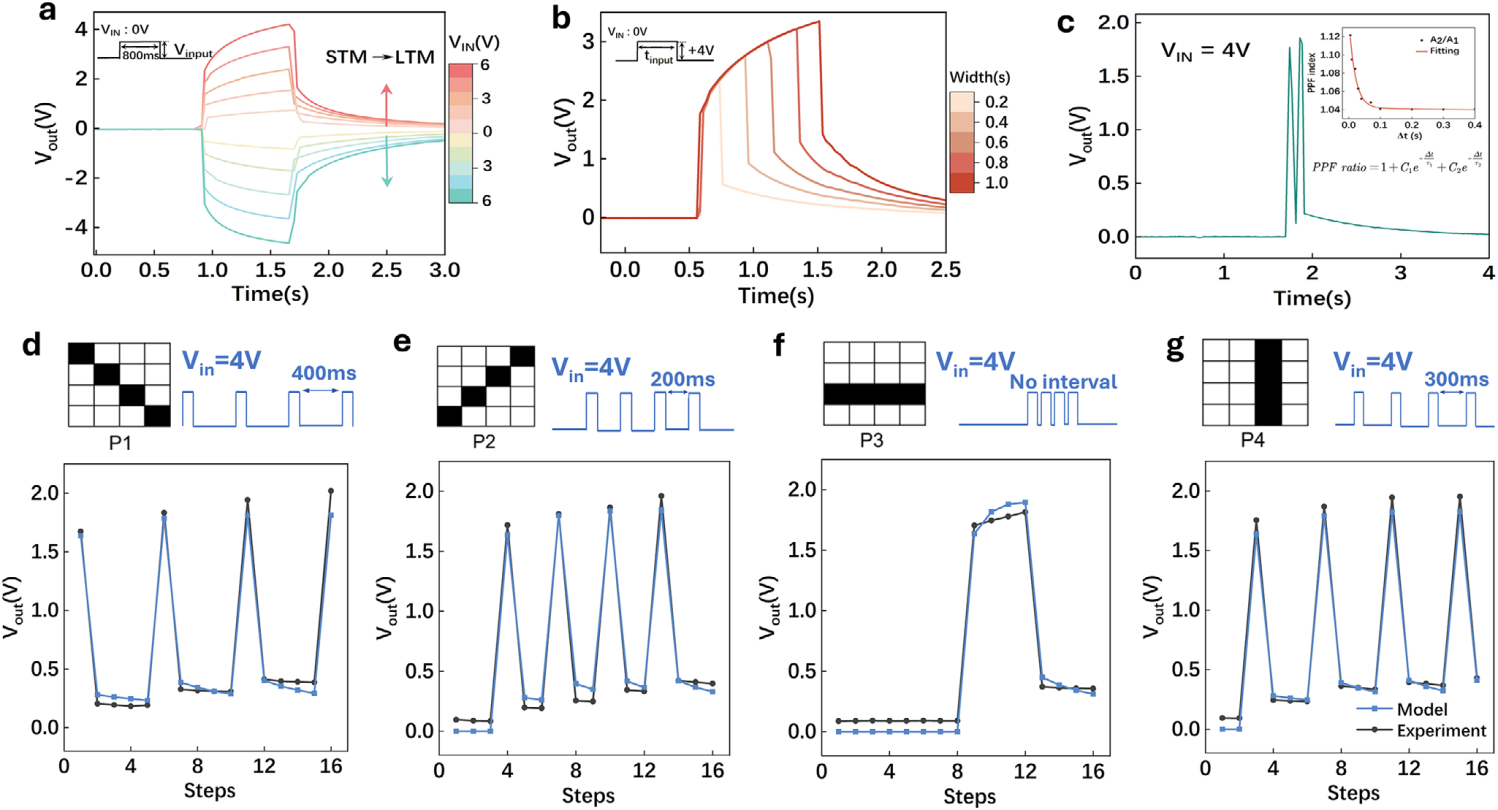
Emulation of Synaptic Plasticity and Dynamic Modulation in the Ferroelectric Transistor (a) Synaptic response of the BTO/HZO/BTO FEFET as the pulse amplitude varies. (b) Synaptic response when the pulse width varies. (c) Paired‐pulse facilitation (PPF) with V_in_ = 4 V. (d–g) Experimental and theoretical responses of the synaptic device under different pulse sequences. In the schematic pixel patterns, black and white represent 4 and 0 V pulses, respectively, with a pulse width and interval of 100 ms per pixel.

In the device configuration, the top gate (Figure 1a) functions as the presynaptic terminal receiving external voltage stimuli, while the bottom gate emulates the postsynaptic response. The synaptic weight update mechanism is realized by modulating the channel conductance through ferroelectric polarization switching in the HZO layer under the applied gate voltage. As shown in Figure 2a, by tuning the V_in_ amplitude at a fixed 800 ms pulse width, the induced V_out_ increases almost linearly with a proportionality of ∼0.67, consistent with prior reports [39, 40].

The synaptic dynamics were further evaluated by varying the pulse width of V_in_ (Figure 2b). The source terminal acts as the presynaptic neuron, while the gate terminal operates as the postsynaptic neuron. The applied pulse waveform serves as the presynaptic trigger, modulating the interfacial surface potential. The resting state is maintained at 0 V to preclude prior charge buildup. As the pulse amplitude increases from 1 to 5 V (0.8 s width), the system exhibits a short‐term memory (STM) response during excitation, followed by a transition toward long‐term memory (LTM) during relaxation, signifying a shift from short‐term plasticity (STP) to long‐term plasticity (LTP) (Figure 2a). Increasing the pulse width from 0.2 s to 1.0 s at 4 V amplifies the storage‐capacitor output and further enhances synaptic plasticity (Figure 2b). This tunable polarization modulation enables controllable nonlinear voltage evolution, providing the high‐dimensional state richness essential for RC frameworks.

As a core indicator of STP, the paired‐pulse facilitation (PPF) quantitatively reflects the device's temporal information‐processing capability. The PPF behavior of the FEFET is presented in Figure 2c. When two identical stimulation pulses (pulse width = 50 ms) are applied with an inter‐pulse interval Δt = 50 ms, the ratio of the second excitatory postsynaptic potential (A_2_) to the first (A_1_) follows a double‐exponential decay with respect to Δt

(1) $$PPF\;Ratio=1+C_{1}exp\left(-\frac{\Delta t}{\tau^{1}}\right)+C_{2}exp\left(-\frac{\Delta t}{\tau^{2}}\right)$$

where τ_1_ and τ_2_ are the characteristic relaxation times, and C_1_ and C_2_ represent the initial facilitation magnitudes. Curve fitting yields C_1_ = 0.0984, C_2_ = 0.0416, τ_1_ = 0.02 s, and τ_2_ = 9.766 s. The fitted dual time constants indicate a biphasic decay, with the fast component (τ₁) associated with STP and the ultralong component (τ_2_) linked to long‐term plasticity LTP mechanisms. These values (C_1_/C_2_ ≈ 2.37) indicate suitability for high‐speed pulse processing while retaining long‐term charge memory, validating the nonlinear, time‐dependent response of the synaptic device.

The dynamic behavior of the capacitive synaptic device is investigated through experiments together with numerical simulations, as illustrated in Figure 2d–g. Four representative pulse sequences are applied to the fabricated device, and the corresponding synaptic responses are compared with model predictions. In each black‐and‐white pixel pattern of Figure 2d–g, the time duration per pixel is 100 ms; a black pixel corresponds to V_IN_ = 4 V, whereas a white pixel denotes no excitation. The synaptic kernel is simulated using Weiss mean‐field theory combined with an empirical polarization transient‐switching model (see Note S4 in the Supplementary Information). The computed V_OUT_ values match the experimentally measured responses across all tested pulse patterns.

### 2.3 Reservoir Computing for Biosignal Classification

**FIGURE 3:**
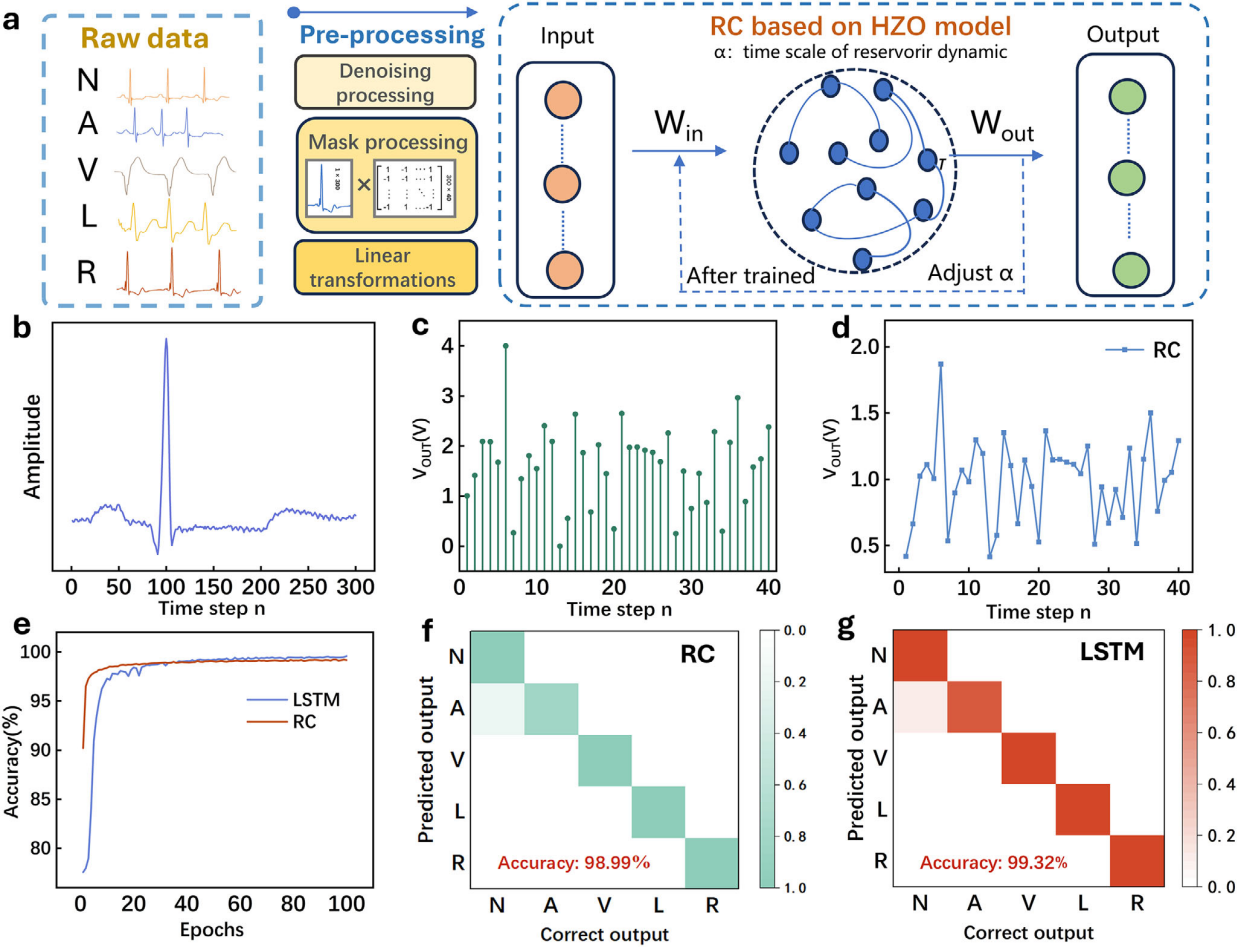
Bio‐Inspired Reservoir Computing for ECG Arrhythmia Classification and Edge‐Health Monitoring. (a) Schematic illustrating the use of the HZO‐based synaptic device for cardiac signal interpretation. (b) Input ECG waveform. (c) Converted voltage input. (d) Output voltage of the synaptic device. (e) Accuracy of LSTM vs. RC. (f) RC accuracy for different arrhythmia subtypes. (g) LSTM accuracy for different arrhythmia subtypes.

The strong consistency between experimental measurements and numerical predictions supports extending the model to evaluate system‐level performance under more complex temporal tasks. Figure 3 presents an example in which the HZO synaptic device is used for electrocardiogram (ECG) interpretation.

ECG is widely used to monitor cardiac electrical activity using wearable platforms such as smartwatches and biosensing patches [41, 42]. These devices are particularly valuable for detecting arrhythmias, a common cardiac disorder.Standard classifications of arrhythmias include normal heartbeat (N), atrial premature beats (A), ventricular premature beats (V), left bundle branch block (L), and right bundle branch block (R) (Figure 3a) [43]. Conventional diagnosis pipelines often rely on recurrent neural network (RNN) or long short‐term memory (LSTM) architectures to achieve high classification accuracy [44]. However, these models are computationally expensive, require large training datasets, and become impractical for low‐power edge hardware [45]. Reservoir computing (RC), as an energy‐efficient RNN variant, is therefore well suited for biosignal processing [46].

The RC architecture comprises an input layer, a nonlinear reservoir layer, and a readout layer (Figure 3a). The reservoir consists of randomly connected nonlinear nodes that project the temporal inputs into a high‐dimensional state space essential for classification [47]. Arrhythmia classification was carried out using the MIT‐BIH arrhythmia database, a widely used benchmark containing long‐duration ECG recordings [48]. The reservoir states are optimized using randomly selected points on the decay curve as reservoir states. These points are mainly used for the inherent charge de‐trapping and depolarization effects of the device to optimize the individual nodes on the ground on the decay curve, instead of simply setting the states to ±1. The raw ECG input signal is linearly converted into a 4 V amplitude voltage signal following wavelet denoising, feature extraction, and decay‐memory‐based masking (see Figure S6 in the Supplementary Information). Each processed input subsequently produces a 40‐point voltage burst, which is used as V_IN_ and fed into the synaptic device through the empirical device model in the simulation. The V_OUT_ signal is then post‐processed via 40‐point interval sampling to generate the final system output for all reservoir states. The dataset is subsequently randomized for cross‐validation of the system model (see Supplementary Material Note S7 for details), and the reservoir states are analyzed using linear regression on the training weights to determine the error between the network output and the target classification labels. Notably, the procedure requires only a single linear regression step throughout the entire workflow, underscoring its simplicity and computational efficiency for lightweight temporal classification tasks. The proposed device model achieves a classification accuracy of 98.99% for ECG data from the MIT‐BIH database (Figure 3f), confirming the excellent data‐processing capability of the BTO/HZO/BTO film‐based energy storage architecture. For comparison, an LSTM model trained under identical conditions (see Note S7 and Note S9 in the Supplementary Materials) achieves 99.32% accuracy (Figure 3g). Although both the LSTM and RC confusion matrices show high recognition accuracy across the five ECG classes, the training time per epoch for the LSTM is substantially longer, and its operational complexity is considerably higher than that of the proposed energy storage model. This efficiency gap originates from the fundamental difference in training paradigms. The RC system requires only a single linear regression step on the reservoir states, while the LSTM relies on iterative backpropagation through time. Our simulations show that this leads to a reduction in training time by over an order of magnitude and a reduction in trainable parameters by more than two orders of magnitude for the RC approach. Complementing this algorithmic efficiency, the synaptic device itself exhibits exceptional energy characteristics. The device operates with an ultralow leakage current of ∼2.5 × 10^−11^ A at 3.0 V(Supplementary Note S5) which yields to an energy consumption of ∼60 fJ per synaptic event, consistent with the literature [46]. The synergy between simplified algorithmic training and inherently low hardware energy consumption establishes the BTO/HZO/BTO‐based synaptic platform as a highly promising candidate for resource‐constrained, low‐power edge computing applications.

### 2.4 Reservoir Computing for Image Recognition

To further validate applicability of the flexible HZO synaptic device in visual perception tasks, handwritten digit recognition was performed using the Modified National Institute of Standards and Technology (MNIST) dataset. The original dataset contains 70,000 black‐and‐white handwritten digit images, each sized 128 × 128 pixels, representing the digits 0–9. After normalization and resizing, the images are converted into 28 × 28 grayscale format containing 784 pixels (see Note S8 in the Supplementary Materials). This representation is widely used for benchmarking hardware‐efficient computing architectures and is ideal for evaluating low‐power synaptic devices.

**FIGURE 4:**
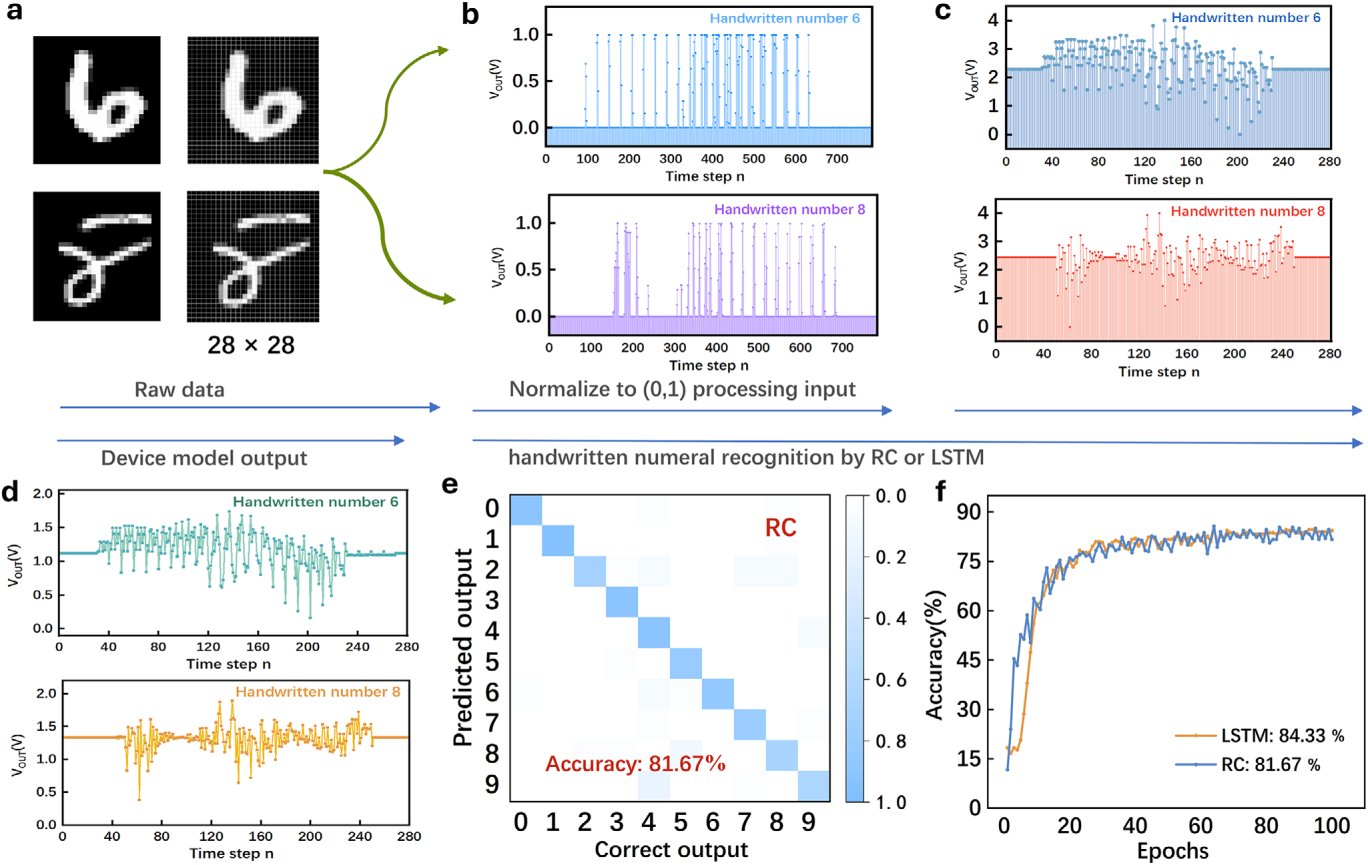
Visual Pattern Recognition via Reservoir Computing on the MNIST Dataset. (a) Example 28 × 28 grayscale images of handwritten digits 6 and 8. (b) Corresponding voltage inputs normalized to 0–1 V. (c) Linear transformation of the inputs.(d) Output responses from the HZO‐based synaptic device. (e) Recognition accuracy of RC vs. LSTM.

During verification, handwritten digits 6 and 8 were randomly selected to visually illustrate the preprocessing workflow. First, the original 128 × 128 grayscale images were converted into 28 × 28 grayscale format while preserving the handwritten proportions and centering the digit within the frame (Figure 4a). The pixel values were then normalized to the range (0, 1) and mapped to pulse sequences, where distinct grayscale intensities generate diverse signal states (Figure 4b). These normalized values were subsequently transformed into 4 V voltage signals via linear scaling. After multiplication with a mask matrix of size 784 × 280, each image produced a voltage pulse train of 280 points (pulse width = 50 ms, pulse interval = 50 ms) (Figure 4c). The preprocessed signal was then applied to the HZO‐based synaptic device model as V_in_, and following post‐processing using ML (ML = 280) interval sampling, the resulting V_out_ values were collected as the final reservoir states. Each training epoch updates the effective reservoir representation by leveraging the intrinsic charge trapping and depolarization behavior of the device.

In handwritten digit classification on the MNIST dataset, the device‐based model achieved an accuracy of 81.67% on the test set (Figure 4e), demonstrating efficient image‐processing capability. Under identical preprocessing and using a two‐layer fully connected readout for comparison, a conventional LSTM model (Figure S7) reached 84.33% accuracy (Figure 4f), as expected. Although both approaches exhibit comparable recognition rates, the LSTM requires significantly longer processing time, whereas the device‐based reservoir model executes the computation more rapidly. This faster response highlights its advantage in feature extraction under high‐throughput conditions, improving the data‐handling efficiency of downstream neural networks.

## 3 Conclusion

To summarize, a freestanding BTO/HZO/BTO film was successfully synthesized using PLD via a water‐assisted exfoliation technique that utilizes the rapid dissolution of an SAO sacrificial layer and the multifunctional BTO buffers. High‐resolution electron microscopy, X‐ray diffraction, and electrical transport measurements confirm high sample crystallinity and stable ferroelectric switching. The freestanding film is prepared using the water‐soluble SAO sacrificial layer and can be transferred onto rigid or flexible substrates, as well as integrated with other 2D material systems. A BTO/HZO/BTO FEFET was fabricated to demonstrate its neuromorphic functionality, exhibiting robust ferroelectricity down to millivolt operation. System‐level simulations confirm that the simplified RC training, combined with the device's low energy consumption (∼60 fJ/event), achieves high classification accuracy with drastically lower computational overhead than LSTM networks. On the other hand, the dynamics of the BTO/HZO/BTO may need further examination for future study. The results in this work advance the convergence of flexible electronics with oxide ferroelectrics and open a pathway toward high‐performance energy‐efficient synaptic devices based on transferable oxide heterostructures.

## 4 Materials and Methods

### 4.1 Target Preparation and Thin Film Deposition

SAO targets were fabricated by mixing SrCO_3_ and Al_2_O_3_ powders via conventional solid‐state synthesis followed by sintering at 1300°C for 24 h. STO substrates were surface‐treated using HF and annealed at 1050°C to ensure single‐layer TiO_2_ termination before epitaxial growth.

### 4.2 Film Deposition

HZO films (40 nm thick) capped by a 10 nm BTO buffer layer were deposited onto (110)‐oriented SrTiO_3_ substrates using a pulsed laser deposition (PLD) system equipped with a COMPex 201 KrF excimer laser (λ = 248 nm) from Coherent Inc. The laser fluence was maintained at 2 J/cm^2^ for Sr_4_Al_2_O_7_ and 1.3 J/cm^2^ for Hf_0_․_5_Zr_0_․_5_O_2_, with oxygen partial pressures of 5.7 × 10^−5^ mbar and 0.1 mbar, respectively. BTO growth was carried out at 0.03 mbar oxygen pressure with a fluence of 1.25 J/cm^2^. The pulse rate was 2 Hz for all targets, and the substrate temperature remained at 750°C. Film growth was tracked in situ by RHEED, and samples were cooled to room temperature at 10°C/min under 200 mbar oxygen.

### 4.3 Film Transfer and Device Fabrication

To preserve film integrity, a PDMS protective layer was spin‐coated onto the HZO film before immersion in deionized water to dissolve the SAO layer. The released films were then transferred to SiO_2_/Si, PDMS, or polyethylene terephthalate (PET) substrates. Prior to transfer, SiO_2_/Si substrates were cleaned ultrasonically in acetone, isopropanol, and deionized water for 15 min each. Gold top electrodes (40 nm) were deposited by thermal evaporation and patterned by photolithography with channel dimensions of 400 µm (width) × 80 µm (length).

### 4.4 Sample Characterization and Electrical Measurements

Crystal structure analysis was performed using an X‐ray diffractometer (XRD, Panalytical X'Pert MRD). For cross‐sectional analysis, samples were prepared via the conventional lift‐out method using a focused ion beam scanning electron microscope (FIB‐SEM, Carl Zeiss Crossbeam 550L) operated at 30 kV. Atomic‐scale microstructure, integrated differential phase contrast (iDPC) imaging, and elemental mapping were conducted at 300 kV using a probe‐corrected Thermo Fisher Scientific Themis Z scanning transmission electron microscope (STEM). The system was equipped with a high‐sensitivity energy‐dispersive X‐ray spectroscopy (EDS) detector and a four‐segment DF4 detector. The imaging conditions included a convergence semi‐angle of 25 mrad, with collection semi‐angles of 50–200 mrad for high‐angle annular dark‐field (HAADF) imaging and 8–42 mrad for iDPC. All electrical measurements were performed with a Keithley 4200 source meter in a probe station under ambient conditions.

## Funding

This work is supported by the National Natural Science Foundation of China (grant No. 12574033, 12204068, 12474125, 52172126 and12204523), the Natural Science Foundation of Jiangsu Province (grant No. BK20220616), the China Postdoctoral Science Foundation (grant No. 2024M753261 and 2025T180149), and the Natural Science Research of Jiangsu Higher Education Institutions of China (grant No. 22KJB140001).

## Conflicts of Interest

The authors declare no conflicts of interest.

## Supporting information




**Supporting File**: advs74068‐sup‐0001‐SuppMat.docx.

## Data Availability

The data that support the findings of this study are available from the corresponding author upon reasonable request.

## References

[1] T. Someya , Z. Bao , and G. G. Malliaras , “The Rise of Plastic Bioelectronics,” Nature 540 (2016): 379–385. 27974769 10.1038/nature21004

[2] T. Someya , T. Sekitani , S. Iba , Y. Kato , H. Kawaguchi , and T. Sakurai , “A Large‐area, Flexible Pressure Sensor Matrix with Organic Field‐effect Transistors for Artificial Skin Applications,” Proceedings of the National Academy of Sciences 101 (2004): 9966–9970. 10.1073/pnas.0401918101 PMC454198 15226508

[3] K. Liu , B. Ouyang , X. Guo , Y. Guo , and Y. Liu , “Advances in flexible organic field‐effect transistors and their applications for flexible electronics,” npj Flex Electron 6 (2022): 1.

[4] Q. Xie , et al., “Flexible Silk‐Fibroin‐Based Electroluminescent Fiber with External‐Field‐Driven Touch Response and Triboelectric Sensing for Smart Wearables,” Advanced Functional Materials (2025): e14650.

[5] Y. Liu , K. He , G. Chen , W. R. Leow , and X. Chen , “Nature‐inspired Structural Materials for Flexible Electronic Devices,” Chemical Reviews 117 (2017): 12893–12941. 28991450 10.1021/acs.chemrev.7b00291

[6] Y. S. Rim , S.‐H. Bae , H. Chen , N. De Marco , and Y. Yang , “Recent Progress in Materials and Devices toward Printable and Flexible Sensors,” Advanced Materials 28 (2016): 4415–4440. 26898945 10.1002/adma.201505118

[7] X. Fu , L. Xu , J. Li , X. Sun , and H. Peng , “Flexible Solar Cells Based on Carbon Nanomaterials,” Carbon 139 (2018): 1063–1073.

[8] Y. Sun and J. A. Rogers , “Inorganic Semiconductors for Flexible Electronics,” Advanced Materials 19 (2007): 1897–1916.

[9] G. Dong , S. Li , M. Yao , et al., “Super‐elastic Ferroelectric Single‐crystal Membrane with Continuous Electric Dipole Rotation,” Science 366 (2019): 475–479. 31649196 10.1126/science.aay7221

[10] Y. Guo , B. Peng , G. Lu , et al., “Remarkable Flexibility in Freestanding Single‐crystalline Antiferroelectric PbZrO_3_ Membranes,” Nature Communications 15 (2024): 4414. 10.1038/s41467-024-47419-w PMC11116490 38782889

[11] J. Zhang , T. Lin , A. Wang , et al., “Super‐Tetragonal Sr_4_Al_2_O_7_ as a Sacrificial Layer for High‐Integrity Freestanding Oxide Membranes,” Science 383 (2024): 388–394. 38271502 10.1126/science.adi6620

[12] K. Nomura , H. Ohta , A. Takagi , T. Kamiya , M. Hirano , and H. Hosono , “Room‐Temperature Fabrication of Transparent Flexible Thin‐Film Transistors Using Amorphous Oxide Semiconductors,” Nature 432 (2004): 488–492. 15565150 10.1038/nature03090

[13] Y. Wang , Q. Wang , J. Zhao , et al., “A Robust High‐Performance Electronic Synapse Based on Epitaxial Ferroelectric Hf_0.5_Zr_0.5_O_2_ Films with Uniform Polarization and High Curie Temperature,” Applied Materials Today 29 (2022): 101587.

[14] X.‐F. Luo , et al., “Interface Engineering in Optoelectronic Hafnium Oxide‐based Heterojunction Synaptic Device for Neuromorphic Computing,” Journal of Colloid and Interface Science 704 (2025): 139324. 41151255 10.1016/j.jcis.2025.139324

[15] N. Liu , J. Zhou , Y. Yao , et al., “HfO_2_‐Based Ferroelectric Optoelectronic Memcapacitors,” IEEE Electron Device Letters 44 (2023): 524–527.

[16] J.‐D. Luo , Y.‐Y. Lai , K.‐Y. Hsiang , et al., “Atomic Layer Deposition Plasma‐based Undoped‐HfO_2_ Ferroelectric FETs for Non‐Volatile Memory,” IEEE Electron Device Letters 42 (2021): 1152–1155.

[17] Y. Peng , W. Xiao , G. Zhang , G. Han , Y. Liu , and Y. Hao , “Synaptic Behaviors in Ferroelectric‐Like Field‐Effect Transistors with Ultrathin Amorphous HfO_2_ Film,” Nanoscale Research Letters 17 (2022): 17. 35072820 10.1186/s11671-022-03655-x PMC8787020

[18] C. Wang , et al., “Bridging Machine Learning and Neuroscience with a Silicon Nanosheet Neuromorphic Device,” Cell Reports Physical Science 6 (2025): 102915.

[19] M. Ahmad , H. Kim , I. Ahmad , et al., “Interface‐Driven Performance Boost in NbO_x_/V_2_O_5_ Bilayer Memristors for next‐generation Neuromorphic Systems,” Materials Today Nano 32 (2025): 100706.

[20] I. Tzouvadaki , P. Gkoupidenis , S. Vassanelli , S. Wang , and T. Prodromakis , “Interfacing Biology and Electronics with Memristive Materials,” Advanced Materials 35 (2023): 2210035. 10.1002/adma.202210035 36829290

[21] L. Václavek , et al., “Mechanical and Optical Properties of HfO_2_ Thin Films Prepared by Evaporation with Ion‐Assisted Deposition,” Materials Today Communications 49 (2025): 114125.

[22] A. Kruv , “Impact of Mechanical Strain on Wakeup of HfO_2_ Ferroelectric Memory,” in 2021 IEEE International Reliability Physics Symposium (IRPS) (2021), 1–6.

[23] W. Banerjee , A. Kashir , and S. Kamba , “Hafnium Oxide (HfO_2_ ) – A Multifunctional Oxide: A Review on the Prospect and Challenges of Hafnium Oxide in Resistive Switching and Ferroelectric Memories,” Small 18 (2022): 2107575. 10.1002/smll.202107575 35510954

[24] H. Zhong , M. Li , Q. Zhang , et al., “Large‐Scale Hf 0.5 Zr 0.5 O_2_ Membranes with Robust Ferroelectricity,” Advanced Materials 34 (2022): 2109889. 10.1002/adma.202109889 35397192

[25] T. Song , V. Lenzi , J. P. B. Silva , L. Marques , I. Fina , and F. Sánchez , “Disentangling Stress and Strain Effects in Ferroelectric HfO_2_ ,” Applied Physics Reviews 10 (2023): 041415.

[26] J. Acker , T. Koschwitz , B. Meinel , R. Heinemann , and C. Blocks , “HF/HNO_3_ Etching of the Saw Damage,” Energy Procedia 38 (2013): 223–233.

[27] M. Badillo , S. Taleb , T. Mokabber , et al., “Low‐Toxicity Chemical Solution Deposition of Ferroelectric ca:HfO_2_ ,” Journal of Materials Chemistry C 11 (2023): 1119–1133.

[28] K.‐I. Park , M. Lee , Y. Liu , et al., “Flexible Nanocomposite Generator Made of BaTiO_3_ Nanoparticles and Graphitic Carbons,” Advanced Materials 24 (2012): 2999–3004. 22549998 10.1002/adma.201200105

[29] T. Tobase , et al., “Pre‐transitional Behavior in Tetragonal to Cubic Phase Transition in HfO_2_ Revealed by High Temperature Diffraction Experiments,” physica status solidi (b) 255, (2018): 1800090.

[30] C. E. Curtis , L. M. Doney , and J. R. Johnson , “Some Properties of Hafnium Oxide, Hafnium Silicate, Calcium Hafnate, and Hafnium Carbide,” Journal of the American Ceramic Society 37 (1954): 458–465.

[31] O. Ohtaka , H. Fukui , T. Kunisada , et al., “Phase Relations and Volume Changes of hafnia under High Pressure and High Temperature,” Journal of the American Ceramic Society 84 (2001): 1369–1373.

[32] H. Cho , P. Pujar , M. Choi , et al., “Expeditiously Crystallized Pure Orthorhombic‐Hf0.5Zr0.5O_2_ for Negative Capacitance Field Effect Transistors,” ACS Applied Materials, Interfaces 13 (2021): 60250–60260. 34894665 10.1021/acsami.1c21387

[33] F. Mattelaer , K. Geryl , G. Rampelberg , T. Dobbelaere , J. Dendooven , and C. Detavernier , “Atomic Layer Deposition of Vanadium Oxides for Thin‐film Lithium‐ion Battery Applications,” RSC Advances 6 (2016): 114658–114665.

[34] R. Materlik , C. Künneth , and A. Kersch , “The Origin of Ferroelectricity in Hf_1−x_Zr_x_O_2_: A Computational Investigation and a Surface Energy Model,” Journal of Applied Physics 117 (2015): 134109.

[35] J. Müller , et al., “Ferroelectricity in Simple Binary ZrO_2_ and HfO_2_ ,” Nano Letters 12 (2012): 4318–4323. 22812909 10.1021/nl302049k

[36] J. Müller , et al., “Ferroelectricity in Yttrium‐Doped Hafnium Oxide,” Journal of Applied Physics 110 (2011): 114113.

[37] K. Hond , G. Rijnders , and G. Koster , “Synthesis of Rhombohedral Hf0.5Zr0.5O_2_ and Analysis by X‐Ray Diffraction through Dynamical Diffraction Simulations,” Materials Advances 5 (2024): 7342–7348.

[38] Q. Hu , S. Lv , C. Xue , et al., “Phase Stability and Phase Transition Pathways in the Rhombohedral Phase of HfO_2_ ,” The Journal of Physical Chemistry Letters 15 (2024): 9319–9325. 39235872 10.1021/acs.jpclett.4c02019

[39] T. Tong , et al., “Reconfigurable Dielectric Engineered WSe_2_/HZO Mem‐Transistor,” 2D Materials 11 (2024): 045012.

[40] W. Kho , et al., “Maximizing the Synaptic Efficiency of Ferroelectric Tunnel Junction Devices Using a Switching Mechanism Hidden in an Identical Pulse Programming Learning Scheme,” Advanced Intelligent Systems 6 (2024): 2400211.

[41] Z. Xue , Y. Gai , Y. Wu , Z. Liu , and Z. Li , “Wearable Mechanical and Electrochemical Sensors for Real‐time Health Monitoring,” Communications Materials 5 (2024): 1–7.

[42] B. Khan , et al., “Electrospun Nanofibers for Wearable Cardiovascular Health Monitoring,” Journal of Science: Advanced Materials and Devices 10 (2025): 101030.

[43] N. F. Banks , A. T. Robinson , D. W. Wray , and K. Bunsawat , “Beyond the QT Interval: How QT/RR Hysteresis May Reveal a Sex‐dependent Hidden Risk for Cardiac Arrhythmias,” American Journal of Physiology‐Heart and Circulatory Physiology 329 (2025): H1006–H1009. 40960945 10.1152/ajpheart.00699.2025 PMC12512364

[44] A. Çınar and S. A. Tuncer , “Classification of Normal Sinus Rhythm, Abnormal Arrhythmia and Congestive Heart Failure ECG Signals Using LSTM and Hybrid CNN‐SVM Deep Neural Networks,” Computer Methods in Biomechanics and Biomedical Engineering 24 (2021): 203–214. 32955928 10.1080/10255842.2020.1821192

[45] T. Lei , Y. Zhang , H. Dai , and Y. Artzi , “Simple Recurrent Units for Highly Parallelizable Recurrence,” in Proceedings of the 2018 Conference on Empirical Methods in Natural Language Processing (Association for Computational Linguistics, 2018), 4470–4481.

[46] S. Jiang , J. Sun , M. Pei , et al., “Energy‐Efficient Reservoir Computing Based on Solution‐processed Electrolyte/Ferroelectric Memcapacitive Synapses for Biosignal Classification,” The Journal of Physical Chemistry Letters 15 (2024): 8501–8509. 39133786 10.1021/acs.jpclett.4c01896

[47] M. Pei , Y. Zhu , S. Liu , et al., “Power‐Efficient Multisensory Reservoir Computing Based on Zr‐Doped HfO_2_ Memcapacitive Synapse Arrays,” Advanced Materials 35 (2023): 2305609. 10.1002/adma.202305609 37572299

[48] G. B. Moody and R. G. Mark , “The Impact of the MIT‐BIH Arrhythmia Database,” IEEE Engineering in Medicine and Biology Magazine 20 (2001): 45–50. 11446209 10.1109/51.932724

