# Supplementary material for: Water‐Assisted Exfoliation of HfO2‐Based Membrane for Flexible Robust Ferroelectric Synaptic Transistors
Source: Adv Sci (Weinh). 2026 Jan 28;13(19):e23654. doi: 10.1002/advs.202523654 (PMC13045468; doi:10.1002/advs.202523654)
Supplement: Supplementary file 1 — Supporting File: advs74068‐sup‐0001‐SuppMat.docx. [file ADVS-13-e23654-s001.docx]

Supporting Information

Water-Assisted Exfoliation of HfO_2_-based Membrane for Flexible Robust Ferroelectric Synaptic Transistors

Han Zhang^1,2^, Miao Zeng^1^, Jingxin Chen^2^, Lei Wang^3^, Sai Jiang^1^, Qian Cao^1^, Xuebo Li^1^, Zekun Hou^1^, Chun Shen^1^，Yulin Gan ^3^, Ning Fang^1^, Zhaoliang Liao ^3*^, Ziyao Zhou^1*^ and Lin Hao^2*^

Han Zhang, Miao Zeng, Sai Jiang, Qian Cao, Xuebo Li, Zekun Hou, Chun Shen, Ning Fang, Ziyao Zhou^*^

The Materials and Electronics Research Center, Changzhou University; Changzhou, Jiangsu, 213159, China

E-mail: ziyaozhou@xjtu.edu.cn

Han Zhang, Jingxin Chen, Lin Hao^*^

Anhui Provincial Key Laboratory of Low-Energy Quantum Materials and Devices, High Magnetic Field Laboratory, HFIPS, Chinese Academy of Sciences; Hefei, Anhui, 230031, China

E-mail: [haolin@hmfl.ac.cn](mailto:haolin@hmfl.ac.cn)

Lei Wang, Yulin Gan^*^ , Zhaoliang Liao^*^

National Synchrotron Radiation Laboratory, School of Nuclear Science and Technology, University of Science and Technology of China; Hefei, 230029, China

E-mail: [ylgan@ustc.edu.cn](mailto:ylgan@ustc.edu.cn)

Note #1

BaTiO_3_ Buffer Layer

The Hf_0.5_Zr_0.5_O_2_(HZO) crystallized in multiple meta-stable structure phases including non-polar ones such as the monoclinic (m-HZO), tetragonal and cubic phase; as well as polar states of the rhombohedral (r-HZO) and orthorhombic (o-HZO) phase. However, it has been established that HZO tends to stabilize in the non-polar states when been deposited on a substrate that has small lattice mismatch to HZO’s pseudo-cubic cell^1^. One way to tackle the problem is to introduce large lattice mismatch so that HZO may be epitaxially grown along its pseudo-cubic [111] direction to form the polar structure. This is the case in this study where HZO was deposited on top of the Sr_4_Al_2_O_7_ (SAO) sacrificial layer. X-ray diffraction (XRD) pattern reveals it to be in the rhombohedral form (Figure S1a). The o-HZO rests on an extensive research foundation and exhibits well-established reliability^2^. In contrast, while advanced material engineering has enabled the realization of promising ferroelectric performance in the r-HZO, its practical viability is conditional on such specialized stabilization methods, and a thorough evaluation of its general device-level reliability remains necessary^3^. Therefore, a BaTiO₃ (BTO) buffer layers were inserted between the SAO and HZO to introduce a larger lattice mismatch so that the ferroelectric o-HZO was seen to be eventually stabilized (Figure S1a red curve).


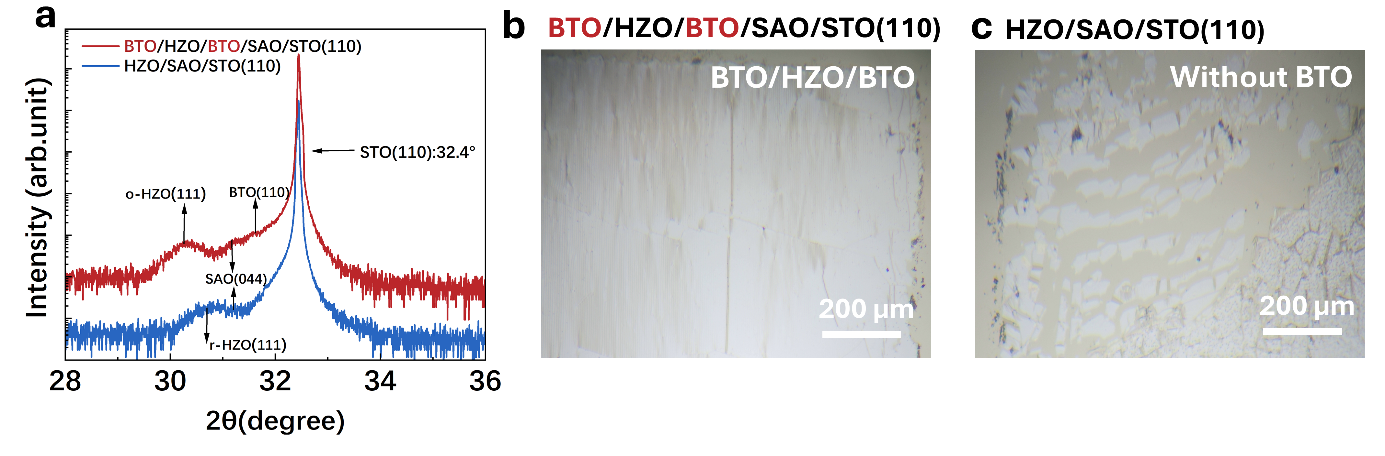


**Figure S1. Effect of the BaTiO_3_ buffer layers.** (a) XRD patterns of BTO/HZO/BTO/SAO/STO(110) and HZO/SAO/STO(110) stacks. (b-c) Optical microscopy images of transferred freestanding membranes from BTO/HZO/BTO/SAO/STO(110), and HZO/SAO/STO(110).

The BTO layer also serves as a mechanical support to extend the flexibility of the membrane. The rapid dissolution of the SAO sacrificial layer generates substantial interfacial stress. The consequences are visually evident in the morphology of transferred membranes: the film from HZO/SAO/STO(110) fragments completely and is untransferable (Figure S1c), whereas the BTO/HZO/BTO heterostructure on STO(110) yields an intact, continuous freestanding membrane (Figure S1b). This mechanical robustness originates from the BTO layers acting as compliant, fracture-resistant matrices—a property consistent with the excellent integrity documented for freestanding BTO membranes.^4,5^

Note #2

Water-Assisted Exfoliation Process via Hydrolysis of the SAO Sacrificial Layer

The fabrication of freestanding BTO/HZO/BTO membranes utilizes a water-assisted exfoliation technique, which is based on the rapid and selective dissolution of a SAO sacrificial layer. SAO dissolves in water at a rate approximately one order of magnitude faster than its conventional cubic polymorph, Sr₃Al₂O₆ (SAO_C_).^6,7^ This significantly enhanced dissolution kinetics originates from its distinct atomic structure: SAO comprises discrete AlO₄⁵⁻ and Al₃O₁₀¹¹⁻ anionic groups, which are more susceptible to hydrolysis than the continuous Al₆O₁₈¹⁸⁻ rings that characterize SAO_C_.^8,9^ The proposed exfoliation process based on this hydrolysis mechanism is schematically illustrated in Figure S2a.

**Figure S2. Mechanism and characterization of water-assisted exfoliation.** (a) Schematic of SAO hydrolysis in water and a photograph of a freestanding BTO/HZO/BTO membrane on PDMS (2×5 mm²), showcasing its macroscopic integrity. (b) XRR data and corresponding fitting curve for the SAO sacrificial layer, confirming its uniform thickness and smooth interface. (c) Optical microscopy images chronologically capturing the release process of a model STO/SAO/STO heterostructure (5 × 5 mm²) in water, demonstrating the generality of the exfoliation method. (d) XRD patterns of the BTO/HZO/BTO heterostructures grown on STO(110) substrates with SAO and SAOc sacrificial layers, respectively.

The effectiveness of the release process is supported by the well-defined properties of the sacrificial layer. X-ray reflectivity (XRR) measurements confirm that the SAO layer has a smooth interface and a uniform thickness of approximately 35 nm (Figure S2b). Due to this structural uniformity and high solubility, the layer dissolves completely within minutes. This rapid and complete detachment process was directly monitored by optical microscopy. The chronological image series in Figure S2c captures the release of a model heterostructure in water, confirming the method’s reliability.

The mild aqueous exfoliation process also preserves the functional properties of the heterostructure. The crystalline phase of the HZO layer is influenced during epitaxial growth by the underlying sacrificial layer. XRD analysis of the as-grown heterostructures (Figure S2d) shows a clear correlation: growth on SAO/STO(110) yields HZO dominated by the ferroelectric orthorhombic (o-) phase, whereas growth on SAOc/STO(110) results primarily in the non-desirable rhombohedral (r-) phase. The subsequent exfoliation successfully transfers this pre-defined functional state, resulting in intact, macroscopic membranes as shown in Figure S3a.

In summary, the combination of a rapidly soluble and structurally uniform SAO sacrificial layer, a reliably observed detachment process, and the preservation of the target ferroelectric phase establishes this water-assisted exfoliation as an effective method for obtaining large-area, transferable functional membranes.

Note #3

Elemental Distribution Analysis by Energy-Dispersive X-Ray Spectroscopy

Elemental mapping and line-scan profiling via energy-dispersive X-ray spectroscopy (EDS) were employed to verify the chemical sharpness and interfacial integrity of the BTO/HZO/BTO heterostructure.


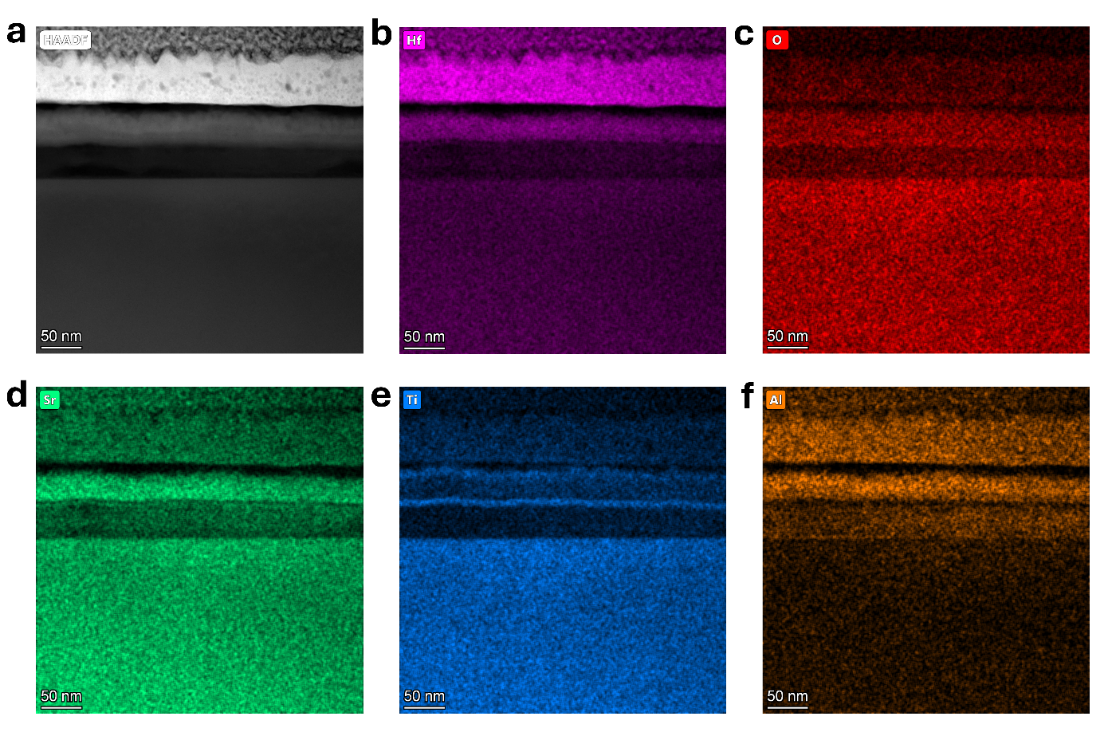


**Figure S3.** EDS analysis of the STO/SAO/BTO/HZO/BTO heterostructure. (a) High-angle annular dark-field (HAADF-STEM) image of the cross-sectional stack. (b-f) Corresponding EDS elemental maps for Hf, O, Sr, Ti and Al, showing well-defined, spatially confined layers without observable interdiffusion.

Note #4

Simulation of Polarization Switching in HZO Memcapacitive Synapses

The dynamic state of the capacitive synaptic core is driven by an applied electric field that breaks the symmetry of the ferroelectric double-well potential, redistributes dipoles, and induces a net polarization shift.^10,11^ Cooperative interactions among dipoles further enhance polarization. The local electric field ($E_{loc}$) on the ferroelectric layer breaks this symmetry, drives dipole redistribution, and induces a polarization change ($\Delta P$), establishing the foundation for the device's non-volatile storage.

$E_{loc}=E_{IN}+\alpha\cdot P$ (1)

where E_IN_ is the field applied in the ferroelectric layer, $\alpha\cdot P$ is the Weiss field evoked by the polarization $P$. $\alpha$ is a feedback factor describing the interactions between the dipoles. Concurrently, the electric field drives polarization reversal, enabling device logic state switching. As the polarization state ($P$) evolves differently under distinct voltage conditions, its temporal dynamics, $P$($t$), reflect the synaptic dynamic state. The relaxation characteristics of $P$($t$) (e.g., exponential decay or dual time constants) directly map to synaptic short-term and long-term plasticity (STP/LTP), providing a physical basis for neuromorphic computing. The transient polarization intensity $P$ is determined by the input voltage V_IN_ and the prior polarization state

$P=nptanh(\frac{p}{kT}\{\frac{V_{IN}}{2d_{fe}}+\alpha\cdot P'\})$ (2)

where n is dipole density, p the dipole moment, k the Boltzmann constant, and $T$ the temperature. The initial polarization state $P'$=–0.6which is chosen empirically to well match the model with experimental measurements. The output potential $V_{IN}$ depends on $V_{IN}$ and $P$, but is also modulated by interface effects such as vacancy migration, charge trapping). Introducing an empirical parameter $k_{1}$ and $k_{2}$ to account for these interface effects results in the final output state:

$V_{OUT}=\frac{V_{IN}}{2k_{1}}+k_{2}P\cdot\Gamma(t)$ (3)

where $\Gamma(t)$ characterizes the polarization transient response.

Note #5

Additional electrical measurements

The BTO/HZO/BTO memcapacitive synapse demonstrates stable long‑term plasticity under sustained input pulses, as evidenced by its output response to an 800 ms voltage stimulus (Figure S4a), confirming its suitability for reservoir computing systems. To evaluate energy efficiency, the static leakage current between the VIN and GND electrodes was measured under a typical operating bias of 3.0 V (Figure S4b). The current remains on the order of $2.5\times{10}^{-11}$ A, indicating minimal standby power dissipation. Operating at ~3.0 V, the system consumes only about 60 fJ per synaptic event, calculated from $3.0\text{ V}\times2.5\times{10}^{-11}\text{ A}\times800\text{ ms}$.


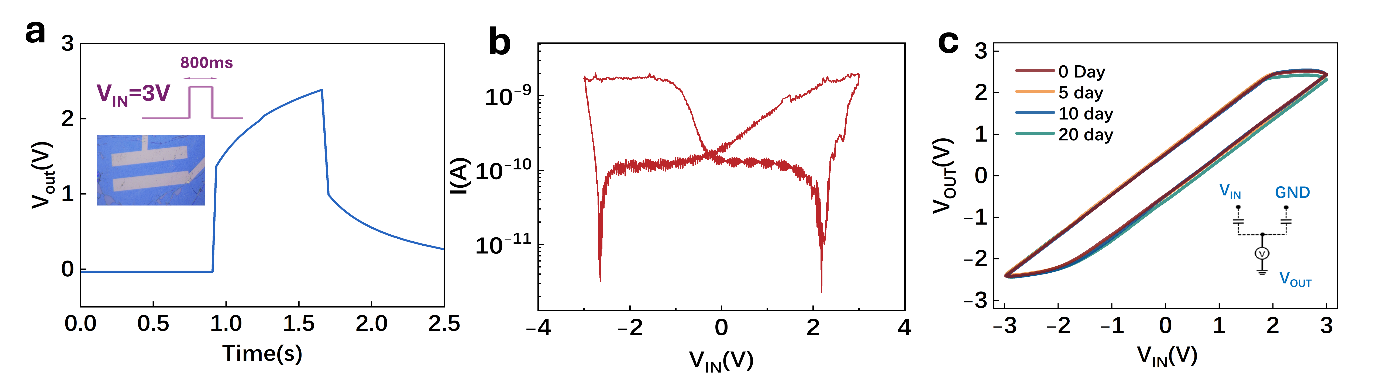


**Figure S4. Electrical characterization of the memcapacitive synaptic device.** (a) V_OUT_ response to an input voltage pulse (800 ms), exhibiting long-term (inset: optical micrograph of the device structure, with electrode dimensions of 400 μm width × 80 μm length). (b) Leakage current between the V_IN_ and GND electrodes at V_IN_ = 3.0 V. (c) Ambient-stability test of synaptic hysteresis. V_IN_-V_OUT_ hysteresis loops measured on a single device after storage at room temperature and pressure for 0, 5, 15, and 20 days.

Beyond static power consumption, the operational stability of the synaptic device under ambient conditions is critical for practical deployment. We monitored the evolution of the core device transfer characteristic, the input-output (V_IN_-V_OUT_) hysteresis, after storage at room temperature and atmospheric pressure. As shown in Figure S4c, the hysteresis curves remain nearly superimposable over 20 days, confirming exceptional stability under ambient conditions.

**Note #6**

**Flexibility Test**

The as-prepared BTO/HZO/BTO membrane was was first transferred to a Indium Tin Oxide/ polyethylene terephthalate (ITO/PET) flexible substrate to test its flexibility. As illustrated below in Figure S5, two gold electrodes are sputtered for electrical measurements, after which the substrate is mounted on a bending apparatus. The surface of the membrane is continously monitored under an optical microscope, with in situ electrical measurments when a voltage of 2V is applied. The results suggest the membrane exhibits no observable fractures with stable current signal down to R=2cm, where R is the bending radius, confirming its flexibility.


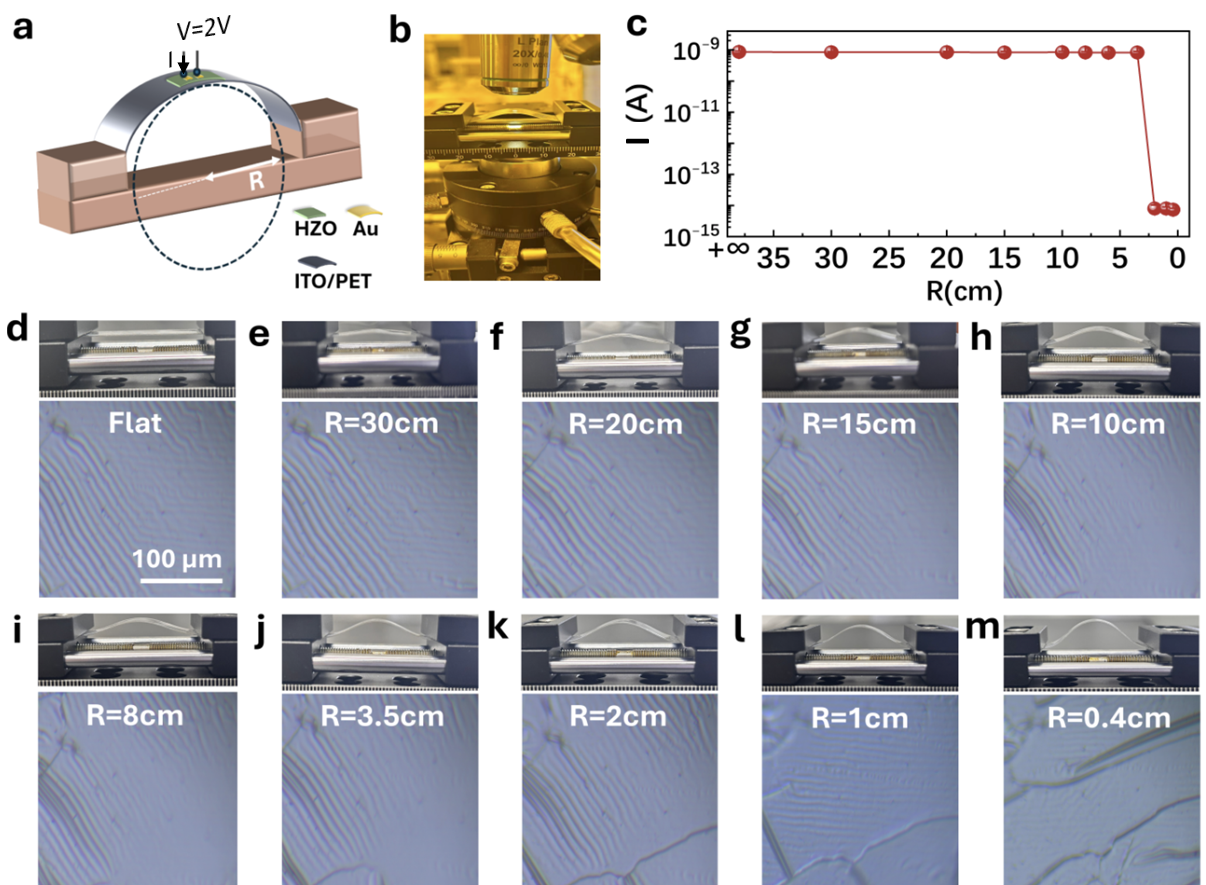


**Figure S5. Flexibility Test**. (a) Schematic drawing of the bending apparatus. (b) A picture of the apparatus. (c) Changes in current under mechanical bending (V_IN_ = 2 V). (d-m) Optical image of the membrane at each bending radius.

Note #7

Preprocessing of Raw ECG Signals from the MIT-BIH Arrhythmia Database

Preprocessing of the raw electrocardiogram signals was carried out through sequential stages of data extraction, denoising, segmentation, and feature compression. Signals and annotations from the MIT-BIH Arrhythmia Database were first parsed to identify sequences of three consecutive heartbeats of identical class to ensure waveform stability. Individual heartbeat segments were extracted using R-wave peaks as temporal anchors, spanning from 99 samples preceding to 201 samples following each R-peak. Wavelet denoising using a ‘db5’ basis with 9-level decomposition and Donoho–Johnstone thresholding was applied to suppress high-frequency noise while preserving morphological fidelity. Each beat was subsequently segmented into 600-sample windows centered on the R-peak, retaining only the five clinically relevant categories (N, A, V, L, R). Feature dimensionality reduction was then implemented via random projection, compressing the 300-sample temporal vectors into a 40-dimensional feature space to improve computational efficiency while preserving discriminatory structure. The final dataset was randomly shuffled and split into training and test partitions according to fixed ratios, forming the basis for subsequent arrhythmia classification.

**
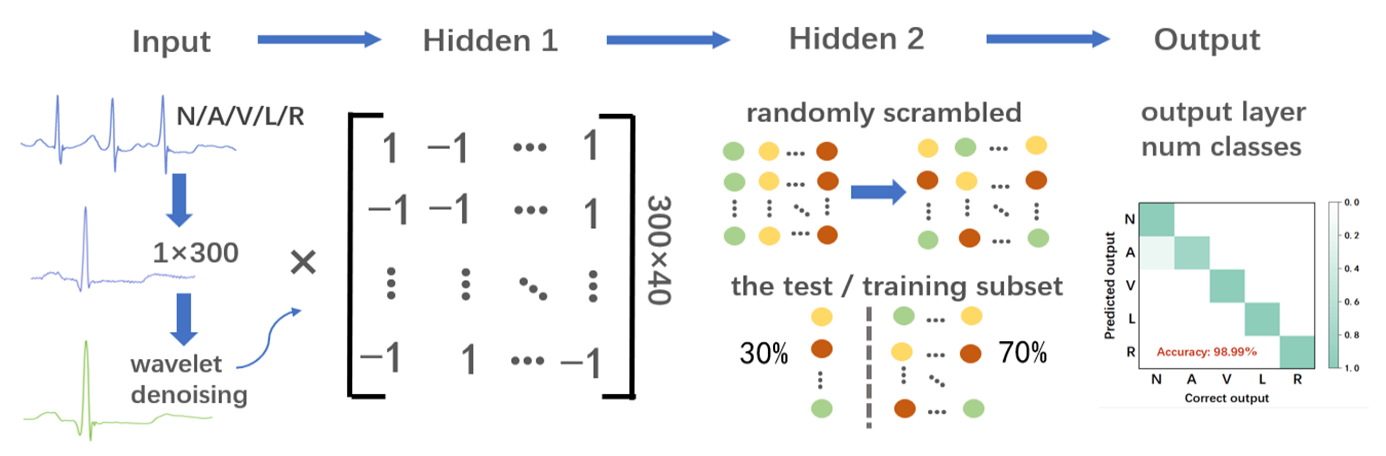
**

**Figure S6.** **Preprocessing of the original ECG signal.**

Note #8

Systematic Preprocessing Pipeline for Handwritten Digit Recognition

Data Preparation and Sampling

The MNIST dataset is decoded from its binary format using custom load_images and load_labels functions, extracting metadata headers and converting raw pixels into (60000 × 28 × 28) image tensors and (60000,) label arrays. To mitigate class imbalance, stratified random sampling (100 samples per digit, 0–9) is applied to create a balanced 1000-sample subset with randomized ordering to avoid temporal bias. Pixel intensities are converted from 8-bit unsigned integers to 32-bit floats and rescaled from [0,255] to [0,1].

Feature Engineering and Transformation

A 28 × 10 random ±1 matrix projects each 28 × 28 image into a 280-dimensional feature vector via inner-product mapping, with tensors reshaped to (1000 × 280). Per-sample min-max scaling normalizes features to a [0,4] range to enhance downstream model stability.

Standardized Data Output

The resulting data are exported to three NumPy arrays: raw_signals.npy (image tensors), labels.npy (digit labels), and pulse.npy (projected features), providing a reproducible and hardware-compatible dataset format for handwritten digit recognition tasks.

Note #9

The LSTM Network Structure

The LSTM network is configured for sequential pattern recognition and contains an input layer (300 temporal steps × 1 feature dimension), followed by a two-layer stacked LSTM block with 128 hidden units per layer and a final fully connected output layer (128 input features, 5 output classes).^12^ The LSTM employs gated memory units — input, forget, and output gates — regulated by sigmoid activation, together with cell and hidden states that maintain long-range temporal dependencies.^13^ Hidden and cell states are initialized as zero vectors at the beginning of each input sequence. The last hidden state is forwarded to the fully connected layer for classification. The network is optimized using cross-entropy loss with the Adam optimizer (learning rate = 0.001, L2 regularization coefficient = 1 × 10^–4^). Model performance is assessed by overall accuracy and confusion matrix evaluation across all arrhythmia classes.


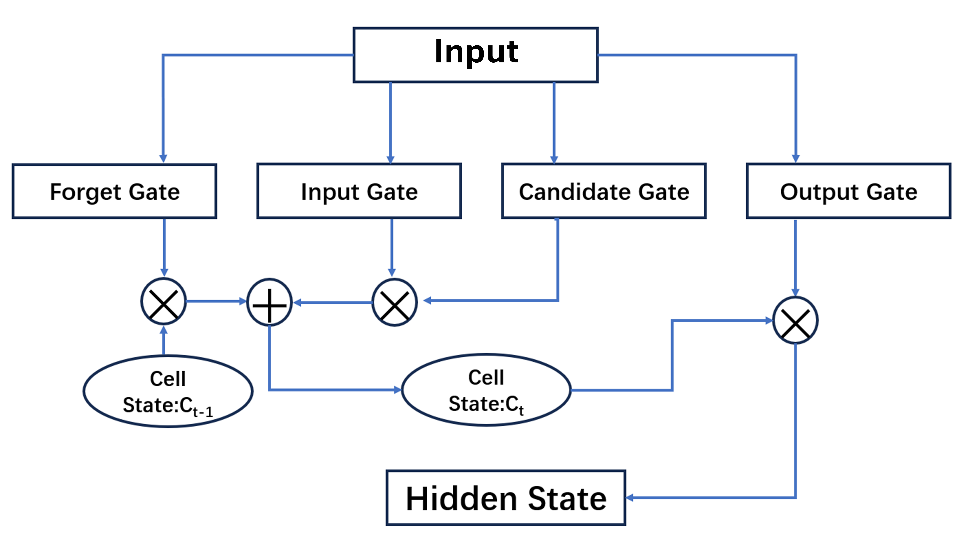


**Figure S7. The LSTM network structure.**

**References**

1. Nukala, P. *et al.* Guidelines for the stabilization of a polar rhombohedral phase in epitaxial Hf_0.5_Zr_0.5_O_2_ thin films. *Ferroelectrics* **569**, 148–163 (2020).

2. Zhong, H. *et al.* Large‐scale Hf_0.5_Zr_0.5_O_2_ membranes with robust ferroelectricity. *Advanced Materials* **34**, 2109889 (2022).

3. Hond, K. de, Rijnders, G. & Koster, G. Synthesis of rhombohedral Hf_0.5_Zr_0.5_O_2_ and analysis by X-ray diffraction through dynamical diffraction simulations. *Mater. Adv.* **5**, 7342–7348 (2024).

4. Dong, G. *et al.* Super-elastic ferroelectric single-crystal membrane with continuous electric dipole rotation. *Science* **366**, 475–479 (2019).

5. Siemenn, A. E. *et al.* Using scalable computer vision to automate high-throughput semiconductor characterization. *Nat Commun* **15**, 4654 (2024).

6. Zhang, J. *et al.* Super-tetragonal Sr_4_Al_2_O_7_ as a sacrificial layer for high-integrity freestanding oxide membranes. *Science* **383**, 388–394 (2024).

7. Nian, L. *et al.* Sr_4_Al_2_O_7_ : A new sacrificial layer with high water dissolution rate for the synthesis of freestanding oxide membranes. *Advanced Materials* **36**, 2307682 (2024).

8. Lu, D. *et al.* Synthesis of freestanding single-crystal perovskite films and heterostructures by etching of sacrificial water-soluble layers. *Nature Mater* **15**, 1255–1260 (2016).

9. Kahlenberg, V., Lazić, B. & Krivovichev, S. V. Tetrastrontium-digalliumoxide (Sr_4_Ga_2_O_7_)—synthesis and crystal structure of a mixed anion strontium gallate related to perovskite. *Journal of Solid State Chemistry* **178**, 1429–1439 (2005).

10. Cheng, L. *et al.* Emulation of synaptic behavior by organic ferroelectric tunnel junctions. *Physics Letters A* **392**, 127138 (2021).

11. Chen, B. *et al.* Ferroelectricity induced double-direction conductance modulation in Hf_x_Zr_1−x_O_2_ capacitors. *Nanotechnology* **33**, 495201 (2022).

12. Yildirim, Ö. A novel wavelet sequence based on deep bidirectional LSTM network model for ECG signal classification. *Computers in Biology and Medicine* **96**, 189–202 (2018).

13. Pulver, A. & Lyu, S. LSTM with working memory. in *2017 International Joint Conference on Neural Networks (IJCNN)* 845–851 (2017).
